# Design and analysis considerations in the Ebola_Tx trial evaluating convalescent plasma in the treatment of Ebola virus disease in Guinea during the 2014–2015 outbreak

**DOI:** 10.1177/1740774515621056

**Published:** 2016-02

**Authors:** Tansy Edwards, Malcolm G Semple, Anja De Weggheleire, Yves Claeys, Maaike De Crop, Joris Menten, Raffaella Ravinetto, Sarah Temmerman, Lutgarde Lynen, Elhadj Ibrahima Bah, Peter G Smith, Johan van Griensven, on behalf of the Ebola_Tx Consortium

**Affiliations:** 1 MRC Tropical Epidemiology Group, London School of Hygiene & Tropical Medicine, London, UK; 2 Institute of Translational Medicine, University of Liverpool, Liverpool, UK; 3 Institute of Tropical Medicine, Antwerp, Belgium; 4 Service des maladies infectieuses et tropicales, de L’Hôpital National Donka, Conakry, Guinea

**Keywords:** Ebola, Guinea, convalescent plasma, trial design

## Abstract

The Ebola virus disease outbreak in 2014–2015 led to a huge caseload with a high case fatality rate. No specific treatments were available beyond supportive care for conditions such as dehydration and shock. Evaluation of treatment with convalescent plasma from Ebola survivors was identified as a priority. We evaluated this intervention in an emergency setting, where randomization was unacceptable. The original trial design was an open-label study comparing patients receiving convalescent plasma and supportive care to patients receiving supportive care alone. The comparison group comprised patients recruited at the start of the trial before convalescent plasma became available, as well as patients presenting during the trial for whom there was insufficient blood group–compatible plasma or no staffing capacity to provide additional transfusions. However, during the trial, convalescent plasma was available to treat all new patients. The design was changed to use a comparator group comprising patients previously treated at the same Ebola treatment center prior to the start of the trial. In the analysis, it was planned to adjust for any differences in prognostic variables between intervention and comparison groups, specifically baseline polymerase chain reaction cycle threshold and age. In addition, adjustment was planned for other potential confounders, identified in the analysis, such as patient presenting symptoms and time to treatment seeking. Because plasma treatment started up to 3 days after diagnosis and we could not define a similar time-point for the comparator group, patients who died before the third day after confirmation of diagnosis were excluded from both intervention and comparison groups in a per-protocol analysis. Some patients received additional experimental treatments soon after plasma treatment, and these were excluded. We also analyzed mortality including all patients from the time of confirmed diagnosis, irrespective of whether those in the trial series actually received plasma, as an intention-to-treat analysis. Per-protocol and intention-to-treat approaches gave similar conclusions. An important caveat in the interpretation of the findings is that it is unlikely that all potential sources of confounding, such as any variation in supportive care over time, were eliminated. Protocols and electronic data capture systems have now been extensively field-tested for emergency evaluation of treatment with convalescent plasma. Ongoing studies seek to quantify the level of neutralizing antibodies in different plasma donations to determine whether this influences the response and survival of treated patients.

## Introduction

At the onset of the outbreak of Ebola virus disease in West Africa in 2014, patients received supportive care (e.g. treatment for dehydration and shock), but no specific treatments had been shown to reduce the very high case fatality rate. Transfusing convalescent whole blood or plasma from patients who had survived and recovered to those with active disease had been a strategy used successfully to treat other infectious diseases.^1,2^ Such blood transfusions were used for eight patients in the Kikwit Ebola outbreak. Although seven survived, it was not clear whether their survival was attributable to the transfusions.^3^ Evaluation of convalescent plasma for the treatment of Ebola disease was identified as a priority by the World Health Organization (WHO) in September 2014,^4,5^ as the treatment, if safe and effective, could be a relatively low-cost intervention that could be scaled up relatively quickly.^6,7^

In September 2014, the Ebola epidemic in West Africa appeared to be out of control, with large numbers of patients presenting for treatment and case fatality rates of around 70%.^8^ Ebola treatment centers were overwhelmed and there were alarming rates of transmission to healthcare staff.^9,10^ The urgency of developing and evaluating better treatments for the disease was recognized by funding agencies and support was made available to study treatments. Several international meetings were held to discuss not only which therapeutic interventions should be prioritized in clinical trials but also the trial design options that would be ethically and scientifically acceptable.^11^ A consortium of European and Guinean collaborators was rapidly formed and a protocol developed to conduct a trial of convalescent plasma in a treatment center run by Médecins Sans Frontières (Doctors without Borders) in Guinea. Funding was awarded for the trial to the “Ebola_Tx” consortium by the European Union on 5 October 2014.

Using the WHO guidance on plasma treatment of Ebola disease,^12^ the protocol for the Ebola_Tx trial sought to balance scientific rigor in design and conduct, with what could be achieved in the challenging field conditions. The trial was designed to be as inclusive as possible so that the design would be ethically acceptable to the concerned community and the results would be widely applicable. Patients of all ages were eligible, including pregnant women and infants. Scientifically, the optimal design would have been some form of randomized controlled trial, preferably blinded, with a parallel arm of patients not receiving convalescent plasma. However, local authorities and field partners in Guinea indicated that a randomized trial including a control arm was unacceptable in the volatile settings of the expanding Ebola outbreak. Another constraint on the design of the trial was that the number of additional blood samples to be taken during the trial was minimized to reduce risks to patients and staff and to ensure that limited clinical resources were not diverted from providing care to patients. As Ebola survivors are a stigmatized and vulnerable population, specific anthropological studies were necessary to investigate attitudes to blood and plasma donations and the use of such donations in treatment.^13–15^

Ethical clearance was sought from the national ethics committee in the study country (Guinea), the Institutional Review Board of the sponsor (Institute of Tropical Medicine, Belgium) the Ethics Committee of the University of Antwerp and those of the collaborating partner institutions—Médecins Sans Frontières, WHO and the London School of Hygiene and Tropical Medicine. The protocol was also reviewed by the scientific commission of the National Ebola Coordination in Guinea. Initial submissions were made on 4 December 2015, and all approvals were obtained by 26 January 2015. The collection of plasma donations from recovered consenting Ebola survivors began on 9 February 2015 and plasma transfusion to Ebola patients on 19 February 2015.

In this article, we discuss challenges in the design and analysis of this trial and several modifications that had to be made to adapt to changing circumstances during the course of the trial. Findings are summarized elsewhere.

## Hypothesis and outcomes of interest

The main objective of the trial was to determine whether treatment with convalescent plasma would reduce the case fatality rate of Ebola patients. All Ebola patients admitted to the treatment center before and during the trial period received supportive care. The trial was designed to have statistical power to detect an absolute risk reduction in mortality of at least 20% associated with plasma treatment. The choice of 20% was debated by international experts during a series of teleconferences organized by WHO. It was considered that a mortality reduction of this magnitude would be required to justify the potential risks to healthcare staff, the significant investment in infrastructure and the commitment of scarce resources that would be necessary to provide widespread access to plasma treatment. Secondary analyses and outcomes included the change in viral load after plasma administration, the correlation of survival with antibody levels in the donor plasma, the incidence of adverse transfusion reactions and any hazards to staff in administering plasma. An overview of the trial is given in Table 1.

**Table 1.** Overview of the Ebola_Tx trial.

Main funder	European Union
Sponsor	Institute of Tropical Medicine, Antwerp
Study site	Conakry, Guinea
Study design/phase	Phase III, non-randomized, comparative study
Inclusion criteria	Confirmed Ebola virus disease—all ages, including pregnant women and infants
Exclusion criteria for receipt of convalescent plasma	History of allergic reaction to blood or blood products
	Medical condition that precludes transfusion (e.g. decompensated heart failure)
	Patients arriving in a close to terminal condition
	Conditions that would jeopardize the safety of treating staff (e.g. agitated patient)
Intervention	Two units of convalescent plasma given consecutively (adults: 2 × 200–250 mL; children: total 10 mL/kg), with each unit from a different donor
	Titers of neutralizing antibodies were not known at time of administration (unselected convalescent plasma)
Primary outcome	Survival at 14 days post administration of convalescent plasma
Secondary outcomes	Survival at 30 days
	Serious adverse reactions
	Change in viral load after convalescent plasma treatment
	Safety risks in health workers
	Risk factors for mortality

## Intervention

In therapeutic drug trials, the dosage of the supposed active ingredient is generally specified and standardized for patients. The presumed mode of action of convalescent plasma is through the presence of neutralizing antibodies in donor plasma. Antibody levels vary from donor to donor; ideally, we would have selected plasma donations with high antibody levels. However, this required shipment of samples abroad to conduct assays for levels of neutralizing antibodies in a high containment laboratory, which entailed long delays. Consequently, we chose to use the WHO-recommended procedure to transfuse two units of 250 mL of plasma not assayed for neutralizing antibodies, each from a different donor, as soon as possible after a confirmed diagnosis of Ebola. All patients in the study were transfused within 3 days of diagnosis.

## Study design

There was much debate about the need for randomization to an experimental treatment or control group to ensure the most scientifically rigorous evaluation. This debate was happening at the same time as expatriates from high-income countries who were infected with Ebola were being evacuated from the epidemic areas and treated in North America and Europe with experimental therapies, outside of controlled trials. In these circumstances, the acceptability of a control arm, in which some patients would receive only supportive care, was challenged for a condition known to have a very high mortality rate. Our own enquiries in Guinea, and those of others, indicated that a randomized trial with a control arm would not be accepted.^16^ We note that a randomized clinical trial was started in Guinea in another Ebola Treatment Unit in July 2015.^17^

**Figure 1.**
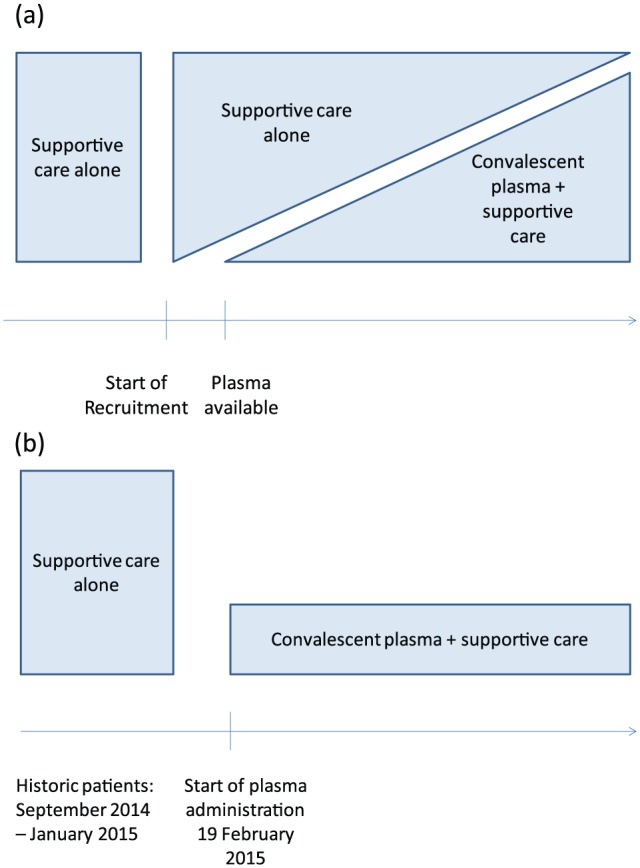
(a) Planned non-randomized design: starting recruitment under the planned non-randomized design was on consideration of adequate plasma becoming available with minimal delay. (b) Implemented design: trial recruitment started once a minimum stock of plasma was available.

The original plan for the trial was a non-randomized parallel group design whereby control patients treated with supportive care alone would be recruited at the start of the trial, while a team worked with survivors and the national blood transfusion service to set up the facilities and to recruit the donors necessary to supply plasma donations. In addition, it was planned that patients not receiving plasma treatment during the trial, because of the lack of blood group–compatible plasma, limited capacity in the treatment center to administer plasma to multiple patients simultaneously or a refusal to receive plasma, would also be enrolled as controls (Figure 1(a)).

## Eligibility for inclusion in the trial, for receipt of plasma and patient prioritization

All confirmed cases presenting at the treatment center were to be assessed for eligibility to receive plasma, provided consent was given, including children and pregnant women. The only patients to be excluded were those with contraindications to plasma treatment, those with very advanced disease (too late to be likely to be influenced by plasma treatment) or agitated patients for whom administration of plasma might be hazardous to staff (Table 1).

During preparation for the study, when the caseload was very high, it was anticipated that operational constraints (insufficient plasma or treatment capacity) would restrict the number of patients who could be treated. Therefore, it was necessary to set up an algorithm to determine which patients would be prioritized for plasma treatment. Allocation had to be done in a way that was perceived as fair to patients and staff but without introducing bias in assessing the impact of the treatment on survival rates. Criteria for prioritization had to be manageable (taking into account the current practices at the laboratory and treatment centers), acceptable (for patients, their family and caregivers) and allow maximal potential benefit of treatment. There was consideration of selecting eligible patients according to their clinical status, such that patients with characteristics known to indicate a poor prognosis would be prioritized. These included patients with signs of shock, pregnant women and children less than 5 years of age. However, this plan was not pursued, as evaluation of plasma treatment would be challenging or impossible with such prioritization, and such selection may have biased against detecting any beneficial effect. “First come, first served” was the preferred approach, after several discussions with the different stakeholders, as the only allocation method accepted as fair. However, if plasma was in short supply, then a queuing system for treatment might have led to patients being treated some days after diagnosis, at a time when plasma might have little effect. Therefore, to avoid accumulating patients on a waiting list, a 48-h window of intervention after diagnosis was added to the prioritization algorithm. For example, suppose Patient 1 was diagnosed on day 1 at 3 p.m., but no compatible plasma was available. Patient 2 was diagnosed on day 2 at 11 a.m. and had the same blood group. If sufficient compatible plasma for one patient became available on day 2 at 4 p.m., Patient 1 would be the one treated. However, if compatible plasma were only to be available on day 3 at 6 p.m. (>48 h after Patient 1's diagnosis), the available plasma would be given to Patient 2.

By the time recruitment began, with the declining epidemic, there was sufficient plasma available to treat all eligible patients and prioritization was unnecessary.

## Sources of comparative data for analysis

Enrolled patients meeting any of the clinical exclusion criteria for plasma treatment (Table 1) received supportive care and were not to be included in comparative analysis. The “control” patients to be included in comparative analysis were those patients who

- Would be eligible for, but refused, plasma treatment;
- Did not receive plasma treatment due a lack of availability of blood group–compatible plasma, either at the start of the study before plasma became available or as recruitment was ongoing once there was a supply of plasma;
- Did not receive plasma due to a lack of clinical capacity to administer the treatment.

A lack of compatible plasma would have been dependent on blood group, which was thought unlikely to be associated with survival and therefore would not introduce bias between comparison groups.

By the time trial recruitment could begin, a large number of willing donors were available and, as Ebola case numbers were falling, the supply of plasma matched the need. Although there was a provision in the original protocol to include data on patients treated at the same treatment center before the start of the trial, to complement concurrent control data in the event that very few control patients following the start of the trial, a protocol amendment was approved to use historic data from patients treated before the trial from the same treatment center as the patients treated with plasma as part of the trial (Figure 1(b)).

## Sample size

When the trial was designed, reports of mortality rates varied substantially between Ebola treatment centers. We did not have information on the rate in the treatment center where the trial would be conducted, and we therefore calculated the required sample size under assumptions of 90% power, a two-sided alpha of 0.05 and an absolute risk reduction of 20%, for a range of mortality rates. We expected that recruitment might have a ratio of 2:1 for plasma-treated and comparator patients, based on caseload reports. Two hundred plasma-treated patients (and 100 comparator patients) would provide 90% power, if mortality in comparator patients was in the region of 40%–80%. In the event of an equal number of plasma-treated and comparator patients being recruited, 130 patients per group would be required to meet the same design assumptions.

As plasma was available for all eligible patients, there were no concurrent control patients who did not receive plasma, and we decided that the most appropriate comparison group would be historic patients treated in the same treatment center from September 2014 until the start of the trial, during which time 507 confirmed cases were admitted.

By July 2015, the Ebola epidemic in Guinea had declined to a low level and few new patients were being treated. On advice from the Data and Safety Monitoring Board, given the decline in the epidemic, recruitment to the study was closed on 7 July 2015. Of a planned recruitment target of 130 patients, 102 patients had been enrolled. Despite having fewer than 130 patients, the high number of comparator patients in the historic series meant that the study was still powered to detect an overall absolute difference in survival of 20%.

## Interim analysis and data and safety monitoring

No interim analysis stopping rule for overwhelming efficacy or futility was included in the trial design. If a benefit of plasma treatment was observed early in the trial, it was planned that plasma administration to new patients would continue as it was of interest to continue data collection to narrow the confidence interval on the effect of the treatment and to gather further data for complementary analyses of antibody titers, changes in viral load and survival. No formal stopping rule for harm was included either, as the Data and Safety Monitoring Board met regularly to review cumulative survival data and serious adverse reaction reports.

The rapidly changing Ebola epidemic and simultaneous evaluation of treatments by different consortia in other Ebola treatment centers meant that the investigators and the Data and Safety Monitoring Board faced many uncertainties. The Board mandate allowed recommendations to stop the trial in various circumstances, such as if the feasibility of plasma treatment became unsustainable due to a shortage of plasma or because of negative perceptions of patients, staff, donors or the local community. The Board was also charged to review the changing environment of the Ebola epidemic in terms of caseload and recruitment rates and the possibility of additional treatment options being shown to be effective in other studies so that they might advise on possible changes to supportive care (e.g. addition of antivirals) and necessary adaptations to the design and sample size.

Importantly, when the trial was started, it was not anticipated that measuring antibodies in donated plasma would be significantly delayed due to administrative and logistic issues. It was recognized that the level of neutralizing antibody levels in donated plasma could not be measured prior to transfusion, but it was anticipated that these would become available later after shipping and laboratory testing in Europe. It was planned that the Data and Safety Monitoring Board would review data on donor plasma antibody levels and patient survival and could potentially recommend a change to the design of the trial, such as restricting plasma donations to those with higher neutralizing antibody levels. These data were not available before the Board recommended an end to trial entry.

Blinding of staff and patients was not possible, but it was planned that investigators and staff would be blind to the summary survival status of patients entered into the trial, although, of course, there was much interest in patient outcomes. This summary information was provided only to the Data and Safety Monitoring Board. Cumulative survival data of patients in the trial and in the historic series were reported to the Board on a regular basis for the primary analysis population. One statistician was unblinded to prepare these results, but investigators remained blind until after the Board recommended to stop recruitment. The only way of keeping staff and investigators blind to the treatment given would have been to have included a control arm in which patients were transfused with plasma not derived from convalescent patients. While this option was considered at the design stage of the trial, it was rejected on largely ethical grounds (but also because of acceptability and logistic issues) in that it was thought there was insufficient scientific evidence that normal plasma would offer benefit and it would subject patients to an unnecessary procedure and staff to the potential hazards of administering the plasma.

## Sources of bias, analytical approach and limitations in context

It is widely acknowledged that a lack of randomization and use of historic controls can lead to treatment groups that are unbalanced with respect to important measured and unmeasured confounding factors that may bias comparative analyses. If, for example, the mortality rate declined during the course of the epidemic, comparison of the survival of recently treated patients with a historic comparison group could lead to a misleading conclusion of benefit of plasma treatment, or an increased mortality risk associated with plasma treatment might be masked.

We considered potential for bias and how to obtain comparable analysis groups in detail (Table 2). We sought to minimize the potential bias in the use of historic controls by adjusting comparative analyses for factors that are known to influence survival, including presenting polymerase chain reaction (PCR) cycle threshold (a measure of viral load) and age. We also examined the variation in mortality rates by month for the period September 2014–January 2015. There was some variation, but our findings were not materially changed by restricting the period for historic controls to November–January.

**Table 2.** Sources of bias, analytical approach and limitations in context using historic controls as the comparison group

Possible source of bias	Problem	Analytical approach in the analysis plan to reduce bias	Implications
*Survival bias*	Definition of a comparable starting point for follow-up for both groups: PCR confirmation of diagnosis may not be received on the same day the sample was taken; Upon confirmation of diagnosis, plasma treatment could be initiated on that day or up to 2 days later; A comparable date for starting plasma treatment could not be defined for historic control patients who were not assessed for eligibility to receive convalescent plasma.	Exclude deaths occurring up to and including the second day after confirmation of Ebola virus disease diagnosis.	Patients in both groups have a comparable starting point of the third calendar day after diagnosis (Figure 2).
			Unbiased comparison of convalescent plasma and historic patients (with respect to follow-up time). Case fatality rate underestimated in both groups as early deaths excluded (Figure 2), but “intention-to-treat” analysis also performed.
	With a starting point of day 3, survival status 14 days post-diagnosis may not allow a full 14 days of follow-up after the onset of treatment in the convalescent plasma group.	The primary outcome was measured as survival status 16 days after diagnosis for both plasma-treated and historic patients, as plasma administration started up to 2 days after diagnosis.	Analysis of the primary endpoint becomes mortality between the 3rd and 16th days post-diagnosis for a harmonized follow-up period. Few deaths from Ebola virus disease were expected after day 16 in either group.
	Patients were discharged once confirmed cured. In the trial, patients were contacted by phone to confirm survival status up to day 30, but this was not done for the historic patients.	Assume historic patients discharged confirmed cured before day 16 were alive atday 16.	It is possible that deaths between days 16 and 30 could be missed for historic patients, but these would likely be due to concomitant conditions or possible sequelae and occur very rarely. One death occurred between days 16 and 30 in a plasma-treated patient after being discharged cured of Ebola disease due to another concomitant condition. Comparison of mortality rates was confined to day 16 only.
		Patients still hospitalized on day 16 are counted as survivors in the primary analysis.	
*Measurement bias and residual confounding*	Accurate data capture was expected for objective factors such as age, sex and PCR cycle threshold as an indicator of viral load.	A priori adjustment for more objective measures of prognostic factors of age and PCR cycle threshold value at diagnosis, based on available literature.	Clinical symptom data may be subjective and there could be variability in reporting in the two periods or missing data for historic series patients if staff had little time for complete data collection and checking. Opportunities may have missed to detect imbalance between groups.
	Although the same variables of interest were being recorded with similar data collection forms by the same on-site team for historic and trial series, there may have been bias in capture of clinical symptom data over time.	Adjust for baseline factors associated with survival in historic patients and also found to be unbalanced between convalescent plasma and historic patient groups.	Effects of confounding taken into account for measured factors, but limitations remain in case of measurement error or reporting bias.
	Patient symptoms and clinical characteristics associated with survival may be imbalanced between treatment groups through a real effect or as a result of reporting bias.		
	Bias toward better survival in trial period, either through better care with additional trial focus on-site or due to lower caseload.		Residual bias may be present.
*Treatment bias*	Supportive care administration could be variable over time.	Although the trial and historic data are from the same Ebola treatment center, managed by the same organization over time, it is not possible to adequately measure or account for variability in supportive care provision in the analysis.	Residual bias may be present.
	Receipt of additional experimental treatments such as Favipiravir would not allow evaluation of convalescent plasma with supportive care versus supportive care alone.	Exclude patients who received additional experimental treatments.	Smaller sample size but more appropriate analysis group.

PCR: polymerase chain reaction.

**Figure 2.**
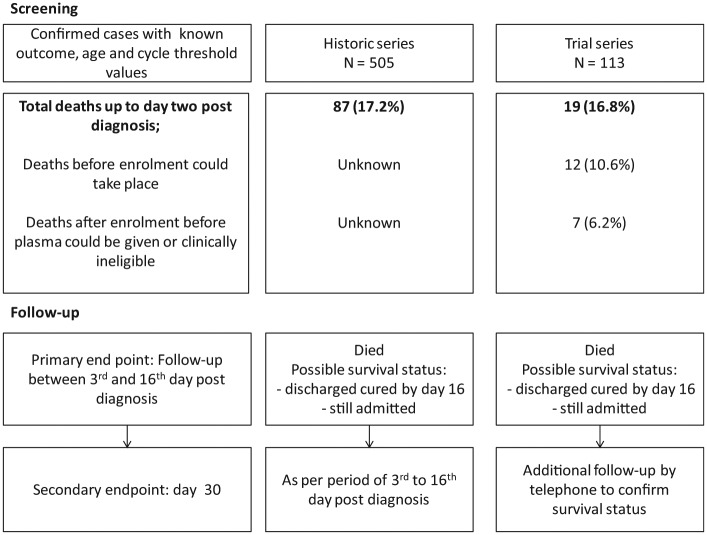
Screening and application of the exclusion criterion of deaths up to 2 days after confirmation of Ebola virus disease diagnosis.

In a randomized trial, the date of start of treatment is clearly defined for each patient. It was not possible to start the administration of plasma immediately after the diagnosis of Ebola, and often there was a delay of a day or, at most, two in initiating plasma treatment. There was no way of identifying a similar time-point for patients in the control series. To overcome this problem, in the comparative analyses of survival, we considered survival from the date of Ebola confirmation plus 2 calendar days and then up to 14 days later in both treated and comparator series (Table 2). Fourteen days of follow-up would then end on day 16 post-diagnosis. In the original protocol, we had not anticipated this issue and had planned to study mortality up to day 14 post-plasma administration. As a consequence, in the primary analysis, we excluded deaths in both series that occurred up to day 3 following diagnosis (Figure 2). This restriction excluded patients who died rapidly after diagnosis, who generally presented with advanced disease, in whom it was considered that plasma was unlikely to influence the course of disease.

We also conducted an intention-to-treat analysis, including all patients in both series from the time of confirmed diagnosis, irrespective of whether patients presenting to the treatment center and confirmed as having Ebola in the trial series actually received plasma.

## Discussion

Randomized controlled trials are the gold standard for assessing new interventions, and the ideal design would have been such a trial, with a control arm in which convalescent plasma was not administered. In a setting where randomized trials of treatment were deemed locally unacceptable, we sought to develop an alternative design while aiming to minimize bias and confounding. We anticipated that we would have a group of patients for whom blood group–compatible plasma was not available and we would also have patients who could not be treated because of other logistic constraints. We considered these patients would be a relatively unbiased comparison group. During the trial, it was possible to treat all patients due to sufficient plasma stocks, so we resorted to using a historic series of patients as the control group. The historic patients had not been assessed for their eligibility for plasma treatment, and thus, it was not possible to apply the same exclusion criteria. However, during the trial, very few patients were excluded from plasma treatment (most of whom died by the third day after diagnosis). In the analyses, we adjusted for measured differences in potential confounding factors, including a measure of presenting viral load. However, in comparing patients treated with plasma to the historic series, it was not possible to be sure that all potential sources of residual confounding had been eliminated, and this is an important caveat in our findings.

In an important complementary analysis, we will seek evidence of any relationship between the antibody level in the donor plasma and the survival and change in viral load in treated patients. These analyses do not need historic data, and treatment with plasma with high or low titers, which was not known at the time of administration, would be expected to be close to random.

Our experience demonstrates that rapidly designing and conducting clinical trials in a humanitarian crisis setting are possible. Substantial time was invested in discussions with the field clinical team on how to prioritize, who to exclude from the trial and how to standardize supportive care. During the inter-epidemic period, such topics should be further discussed to reach a consensus before the next outbreak. During an outbreak, early consultation and involvement of both academic and operational partners are indicated.

The Ebola_Tx protocol, standard operating procedures, case report forms and data management system with electronic case report forms, specifically developed for the high-risk treatment center context,^18^ have now been extensively field-tested and can be adapted for other future outbreaks of hemorrhagic fever where convalescent plasma is a proposed treatment. It is unclear whether randomization to treatments would be acceptable to patients or healthcare workers in the future, but preparedness for collecting standardized control data from all treatment centers could be enhanced by sharing field-tested materials between existing networks (e.g. the International Severe Acute Respiratory and Emerging Infection Consortium, https://isaric.tghn.org/) so that they can be rapidly implemented from the onset of any new outbreak.

The main outstanding challenge to all trials of treatments for Ebola is the lack of standardized protocols for harmonized and stable supportive care, which is important irrespective of the study design chosen. Standardized point of care tools could better inform patient management and improve standardization. High-level, patient-directed care with aggressive management of hypovolemia, electrolyte disturbances and kidney injury, and including antivirals if shown to be effective, might improve supportive care in future therapeutic trials.^19,20^ In-depth anthropological studies should also be conducted to gain a better understanding on community acceptability of randomization during outbreaks of diseases with high case fatality rates.

## Conclusion

Although a randomized trial would have been methodologically preferable, the Ebola_Tx trial represents a valuable experience of an alternative design, developed during an outbreak where randomization was not acceptable. Due reflection and efforts were dedicated on minimizing potential sources of bias in our comparison of mortality among patients receiving supportive care with the addition of treatment with convalescent plasma. We cannot exclude the possibility of residual bias due to the lack of randomization and blinding in the design. Having conducted the largest ever study of convalescent plasma for Ebola disease, field-tested protocols and electronic data capture systems are now available for high-risk emergency evaluation of such treatment. Neutralizing antibody levels in donated plasma will allow further consideration of the effect of the treatment.
